# Biophysical Characterization of the Feline Immunodeficiency Virus p24 Capsid Protein Conformation and *In Vitro* Capsid Assembly

**DOI:** 10.1371/journal.pone.0056424

**Published:** 2013-02-15

**Authors:** Jennifer Serrière, Daphna Fenel, Guy Schoehn, Patrice Gouet, Christophe Guillon

**Affiliations:** 1 Laboratoire de Biocristallographie et Biologie Structurale des Cibles Thérapeutiques, IBCP-BMSSI, UMR 5086 CNRS Université de Lyon, SFR BioSciences Gerland-Lyon Sud, Lyon, France; 2 CEA, Institut de Biologie Structurale Jean-Pierre Ebel, UMR 5075, Grenoble, France; 3 CNRS, Institut de Biologie Structurale Jean-Pierre Ebel, UMR 5075, Grenoble, France; 4 Université Joseph Fourier, Institut de Biologie Structurale Jean-Pierre Ebel, UMR 5075, Grenoble, France; 5 Unit for Virus Host Cell Interactions, UMI 3265-CNRS-EMBL-UJF, Grenoble, France; Centro de Biología Molecular Severo Ochoa (CSIC-UAM), Spain

## Abstract

The Feline Immunodeficiency Virus (FIV) capsid protein p24 oligomerizes to form a closed capsid that protects the viral genome. Because of its crucial role in the virion, FIV p24 is an interesting target for the development of therapeutic strategies, although little is known about its structure and assembly. We defined and optimized a protocol to overexpress recombinant FIV capsid protein in a bacterial system. Circular dichroism and isothermal titration calorimetry experiments showed that the structure of the purified FIV p24 protein was comprised mainly of α-helices. Dynamic light scattering (DLS) and cross-linking experiments demonstrated that p24 was monomeric at low concentration and dimeric at high concentration. We developed a protocol for the *in vitro* assembly of the FIV capsid. As with HIV, an increased ionic strength resulted in FIV p24 assembly *in vitro*. Assembly appeared to be dependent on temperature, salt concentration, and protein concentration. The FIV p24 assembly kinetics was monitored by DLS. A limit end-point diameter suggested assembly into objects of definite shapes. This was confirmed by electron microscopy, where FIV p24 assembled into spherical particles. Comparison of FIV p24 with other retroviral capsid proteins showed that FIV assembly is particular and requires further specific study.

## Introduction

Feline Immunodeficiency Virus (FIV) is the causative agent of feline AIDS and a member of the lentivirus group in the family *Retroviridae* [1]. FIV has been used as an animal model for Human Immunodeficiency Virus (HIV) infection [2], because infections with FIV and HIV share biological and clinical characteristics [2], [3]. FIV induces AIDS in cats and wild felids with high prevalence rates in some geographical areas [3], so the development of effective anti-FIV strategies is of great veterinary interest. No specific antiviral drug for FIV is currently available.

Retroviruses are enveloped viruses that contain a capsid inside the virion that protects the genomic material. This architecture, which is necessary for virus infectivity, has been extensively studied [4]. Initially, this viral capsid assembles into spherical particles that contain approximately 1,500 molecules of the complete Gag polyprotein [5]. In these immature capsids, the Gag polyprotein is arranged radially where the myristylated N-terminus of the matrix domain is anchored in the envelope of the viral particle [6], [7]. After assembly and budding, these immature virions rearrange to form mature infectious viral particles [8]. Viral maturation is initiated by proteolytic cleavage of the Gag polyprotein into subunits including the matrix (MA), capsid (CA), and nucleocapsid (NC), by a viral protease. This results in global rearrangement and new protein/protein interactions within the viral particle. The matrix protein remains associated with the envelope, while the nucleocapsid protein associates with the viral genome. The capsid protein, also known as p24 in HIV or FIV, condenses to form a closed capsid shell of variable shape depending on the viral group [9]. The capsids of lentiviruses have a cone-shaped fullerene geometry [10]–[12] with dimensions of 120 to 130 nm in length, 55 to 62 nm in diameter at the broad end, and 25 nm in diameter at the tapered end. It is estimated that 1,500 p24 proteins, associated in hexamers and pentamers, form this conical shape [13], [14], as observed by transmission electron microscopy (TEM) [10], [11], [15]. The formation of the immature particle and its conversion to a mature virus is sensitive to mutations in the p24 protein, indicating that p24 contains information required at various stages of viral assembly [16].

The formation of the mature viral core is a critical step in the virus life cycle. Only mature viruses are infectious, suggesting a role for a morphologically correct viral capsid in virus infectivity. Assembly appears to be a good target for antivirals because it depends on repetitive weak protein interactions. Disruption of any part of these interactions may be sufficient to suppress the infectivity of HIV [17]. To date, only a few studies have attempted to identify such molecules in HIV-1 and assess their ability to inhibit virus assembly[18]–[23].

Retroviral p24 proteins contain two structural domains that are mainly helical, i.e., the N-terminal assembly domain (NTD) and the C-terminal dimerization domain (CTD). Previous studies of HIV have shown that NTD is essential for mature capsid assembly [24]–[27] while the p24 CTD dimerizes in solution [28], [29]. Mutational analysis has shown that this dimeric CTD interface is essential for infectivity and the assembly of virions [17]. *In vitro* assembly systems were developed to better understand the biological mechanisms of capsid assembly and investigate the efficiency of potential assembly inhibitors, in which purified viral domains can be induced to assemble into biologically relevant structures. Previous studies using purified capsid proteins have provided information on the *in vitro* capsid assembly mechanism [16], [17], [30]. These studies suggest that the recombinant capsid protein alone can undergo multimerization in specific biochemical conditions, leading to the formation of high-order oligomers with a specific morphology, as revealed by electron microscopy. The most abundant high-order oligomers detected in HIV after *in vitro* assembly are cylindrical elongated fibers of variable length [11], [16], [17], [31], while other structures with a conical or spherical appearance were occasionally observed with the same constructs [16]. The different structures obtained after *in vitro* assembly are determined by the experimental conditions, e.g., the pH and/or ionic strength of the reaction mix, as well as by the presence of Gag sequences at the N- or C-terminal end of the HIV capsid protein [32], [33]. The sensitivity of these different structures to experimental factors such as the ionic strength indicates that polar interactions on the multimer surface might regulate their formation. In contrast to HIV, the *in vitro* assembly of Rous Sarcoma Virus (RSV) capsids into regular icosahedral particles under acidic conditions is not induced by an increase in ionic strength [34].

In this study, we purified FIV capsid protein and characterized its assembly *in vitro*. First, we quantitatively monitored *in vitro* FIV capsid assembly by DLS to evaluate the effects of temperature conditions, salt concentration, and protein concentration on FIV capsid assembly. Our DLS approach was combined with TEM analysis to show that the FIV recombinant p24 proteins could form spherical capsid particles.

## Materials and Methods

### Construction of a Plasmid Expressing the FIV p24 Protein

The FIV p24 open reading frame of the Petaluma strain was amplified from plasmid FIV-34TF10, which was obtained through the AIDS Research and Reference Reagent Program, Division of AIDS, NIAID, NIH from J. Elder.

For PCR amplification, we used the forward primer (5′-GGATCCAATAGAAGGACGACCTATTCAAACAGTAAATG-3′, *BamH*I site underlined), and the reverse primer (5′-GAATTCTCAGAGTTGCATTTTATATCCTG-3′, *EcoR*I site underlined). The PCR product was digested with *BamH*I and *EcoR*I then inserted into the prokaryotic expression pRSET-B (Invitrogen), which was digested with the same enzymes, to create a p24 expression plasmid pRSET-p24 encoding p24 with a hexa-histidine tag at the N-terminal extremity of the protein.

### Expression in *Escherichia Coli*

Expression of the FIV capsid sequence was performed in *E. Coli* BL2I(DE3)pLysS (Novagen). Briefly, 1 µg of plasmid DNA was transformed into 50 µl of competent cells. Positive clone cells were grown at 37°C in Turbo Broth medium (AthenaES), supplemented with 25 µg/ml of ampicillin. When cultures reached an OD_600_ value of 0.3–0.4, the expression of capsid protein was induced by the addition of isopropyl-β-D-thiogalactopyranoside (IPTG) to a final concentration of 2 mM. After 20 h of induction at 25°C, cells were collected by centrifugation and the wet pellet was stored at –20°C overnight.

### Purification of FIV Capsid Protein

Recombinant capsid protein was purified by nickel affinity chromatography. Briefly, 10 g of frozen cell pellet was resuspended in 10 ml of LEW buffer (50 mM NaH_2_PO_4_, 300 mM NaCl), pH 8, with lysozyme (Sigma) at a final concentration of 1 mg/ml, and anti-protease cocktail 1× (Halt™ Protease Inhibitor Cocktail, Thermo Scientific) and DNase I (Sigma) at a final concentration of 2 U/ml, followed by 1 h on ice. The resuspended pellet was lysed by sonication during 3×3 min, using a SONIFIER 250 (Branson). The bacterial lysate was clarified by centrifugation at 10,000×*g* for 20 min, before the supernatant was filtered through a 0.45 µm membrane. The supernatant was loaded onto a gravity column packed with 2 g of Ni^2+^-TED resin (Macherey-Nagel) and eluted with LEW buffer containing 50 mM of imidazole after three washes with LEW buffer. His-tagged p24 protein purification was quantified by measuring the absorbance at 280 nm, using a Nanodrop (ThermoFischer). The identity and the purity of the target fusion protein were evaluated using standard SDS-PAGE analysis. According to the SDS-PAGE analysis of the soluble fraction and cell debris pellet, the majority of the induced protein was present in the soluble fraction (data not shown). Protein was dialysed against 50 mM sodium phosphate (pH 7.4) and concentrated using Vivaspin ultrafiltration devices (10 kD MWCO, Sartorius). The absence of DNA in the protein sample was confirmed by Ethidium Bromide stained agarose gel electrophoresis (data not shown).

### FIV Capsid Protein Analysis by Circular Dichroism

CD spectra were recorded using a Chirascan Dichrometer (Applied Photophysics). Experiments were performed at 20°C with 150 µl of protein at 10 µM in 50 mM sodium phosphate (pH 7.4). The measurements were made using a glass cuvette with a 0.1 cm optical path, in the far UV (190–260 nm) with an increment of 0.5 nm and 1 s integration time. The signal due to buffer alone was subtracted from the spectra obtained in the presence of the protein. Spectra were then processed with the CD6 program, i.e., baseline correction, smoothing, and conversion of spectral units into [θ].

The estimated proportions of the secondary structures of proteins were derived from the [θ] values between 190 and 240 nm, using the DichroWeb server (http://dichroweb.cryst.bbk.ac.uk/html/home.shtml) and the SELCON3 algorithm.

### FIV Capsid Protein Analysis by Dynamic Light Scattering

Purified capsid proteins in 50 mM sodium phosphate buffer at pH 7.4 were concentrated to 5 and 7 mg/ml using a Vivaspin centrifugal concentrator (exclusion limit 10,000; Vivaspin, Sartorius). DLS experiments were conducted at 23°C using a Malvern-dynamic light scattering Zetasizer Nano S ZEN1600 (Malvern Instruments). Each measure was the mean of 12 runs and there were three replicates of each experiment.

### Isothermal Titration Calorimetry (ITC) and Estimation of the K_D_

Dilution experiments were conducted to investigate the dissociation process of the dimer-to-monomer transition using an ITC200 calorimeter (GE Healthcare). Protein samples of FIV p24 at 7 mg/ml (243 µM) into 50 mM sodium phosphate pH 7.4 were used. Dilution ITC experiments involved sequential injections of concentrated protein solution at 243 µM (injection #1∶1 µl and injections #2–16∶2.5 µl), spaced at 120 seconds intervals, into the calorimetric cell (200 µl), which initially contained buffer alone (50 mM sodium phosphate pH 7.4). The dilution of the protein solution shifts the equilibrium toward the monomer, and the amount of heat either released or absorbed upon dissociation into monomers can be monitored as a function of the total concentration of the protein in the calorimetric cell. Measurements were performed at 30°C, and data were analyzed using a dissociation model in Origin according to the manufacturer’s instructions (GE Healthcare). The first data point was excluded in the analysis. The binding parameters Δ*H* (reaction enthalpy change in cal mol^−1^) and *K* (dissociation constant in mM) were allowed to float during the fit.

### Chemical Cross-linking

Chemical cross-linking experiments provide a direct method for identifying transient and stable interactions. This technique involves the formation of covalent bonds between proteins using bifunctional reagents that contain reactive end groups, which react with the functional groups of amino acid residues, such as primary amines and sulfhydryls.

Purified p24 protein was mixed with bis(sulfosuccinimidyl) suberate (BS^3^) at different concentrations of p24 (5 and 7 mg/ml). Reaction mixtures with 7 µg of protein were incubated with 1 µl of 5 mM BS^3^ (corresponding to a 20∶1 (w/w) BS^3^:p24 ratio) for 30 min at room temperature. The reaction was stopped by the addition of 1.5 µl 250 mM Tris-HCl (pH 7.5) and 30 mM glycine for 15 min at room temperature. Cross-linked proteins were solubilized by the addition of Laemmli’s sample buffer, before 7 µg of cross-linked protein was transferred to SDS-PAGE.

### Kinetic Analysis of Assembly Reactions by Dynamic Light Scattering

Capsid protein assembly was triggered by changing the NaCl concentration from 0 to 0.5 or 1 M in buffer containing the protein at different concentrations (1∶1 mixture of protein solution at 5 or 7 mg/ml with 1 M or 2 M NaCl solution).

Purified capsid protein in 50 mM sodium phosphate buffer (pH 7.4) and concentrated to 5 or 7 mg/ml was placed into a low-volume disposable glass cuvette (Malvern Instruments). The stability of the particle diameter was measured at 37°C, 30°C, and 25°C, before the addition of NaCl, by DLS for 10 min every 2 min.

Assembly was initiated by the addition of 1 or 2 M NaCl (0.5 or 1 M final) into the cuvette. The reaction was rapidly homogenized and DLS measures were collected every 2 min for 40 min. Approximately 30 s elapsed between the addition and the first time point measurement. In the kinetic analysis, the assembly reaction was tested at final concentrations of 2.5 and 3.5 mg/ml capsid protein.

### Transmission Electron Microscopy

In the TEM analysis, capsid proteins were assembled at a final concentration of 3.5 mg/ml with a final NaCl concentration of 0.5 M, and assembly was monitored by DLS in parallel.

The assembly products were analysed by TEM after different incubation periods (T = 0 min, 4 min, 8 min, 12 min and 60 min) without centrifugation to ensure the observation of assembly intermediates. Five microliters of each sample were adsorbed during 2 minutes on grids coated with colloidal carbon and made hydrophylic by glow-discharge for 1 minute. Grids were washed once in phosphate buffer pH 7.4, and fixed 5 minutes in 0.1% glutaraldehyde (in phosphate buffer). After 2 washes in buffer and 1 wash in water, grids were stained for 30 seconds in a drop of 2% ammonium molybdate and dried before vizualisation using a Phillips CM12 electron microscope working at 120 KV, with a magnification of 45,000×.

## Results

### Expression and Purification of Recombinant p24 Proteins

**Figure 1:**
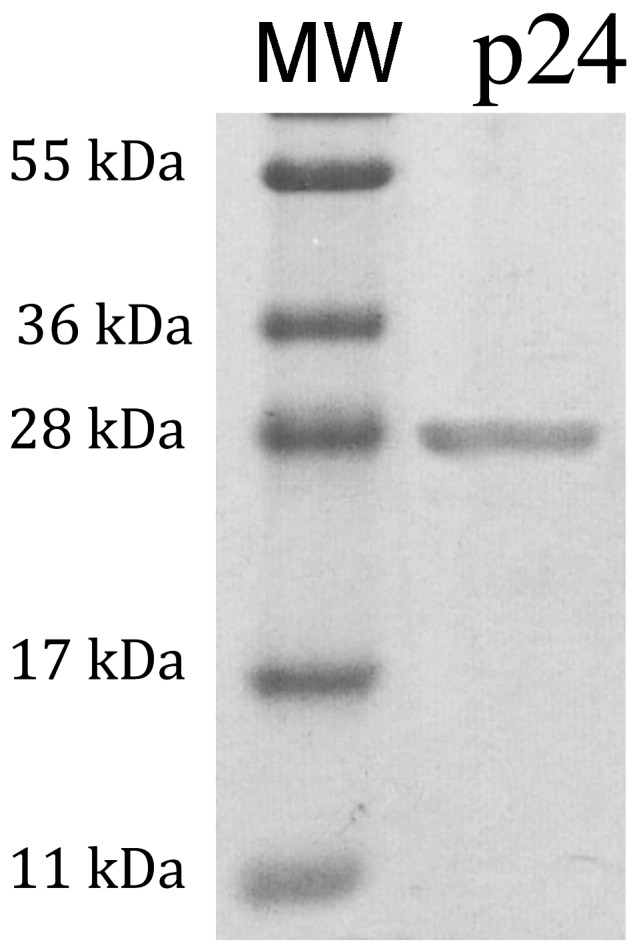
SDS-PAGE analysis of the FIV recombinant p24 protein purity. Lane MW, protein molecular mass marker; Lane p24, concentrated and purified His-tagged p24 protein.

The purity of the protein was verified by SDS-PAGE after purification of the His-tagged p24 protein using a Ni^2+^-TED resin column and concentration (Figure 1). A single band that matched the expected size of the protein (28.5 kDa) was observed, suggesting a protein purity >90%.

### FIV Capsid Protein Analysis by Circular Dichroism

**Figure 2:**
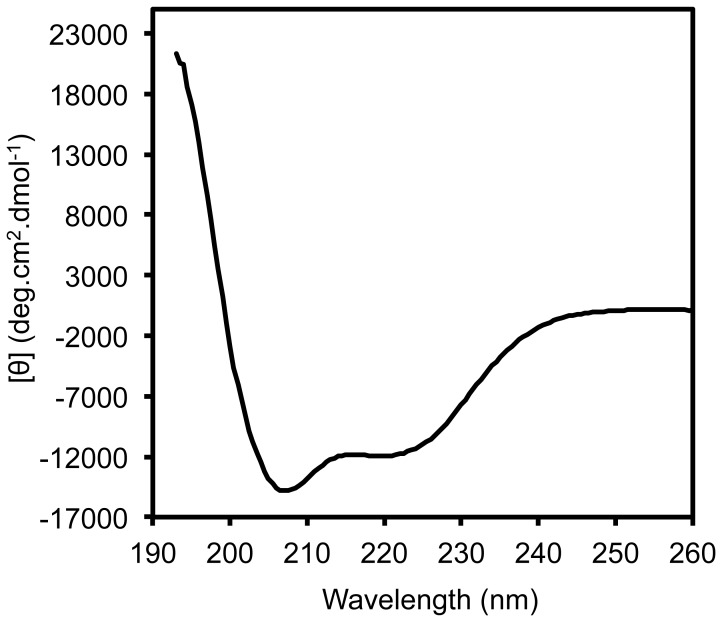
Circular dichroism spectrum in the far-UV for the FIV p24-His protein. Experiments were performed at 20°C with 150 µl of protein at 10 µM in 50 mM sodium phosphate (pH 7.4).

To analyse the folding of the FIV recombinant capsid protein, we used CD to measure the percentage of each type of p24-His secondary structures in solution (Figure 2). The CD spectrum indicated the presence of a maximum at 190 nm and two minima at 208 nm and 220 nm. This was characteristic of a protein structure containing a majority of α helices. The percentage of α helix, β sheet and turns for p24-His protein were estimated using Dichroweb with the SELCON 3 algorithm at 41%, 15% and 17%, respectively.

### FIV Capsid Protein Analysis by Dynamic Light Scattering and Chemical Cross-linking

**Figure 3:**
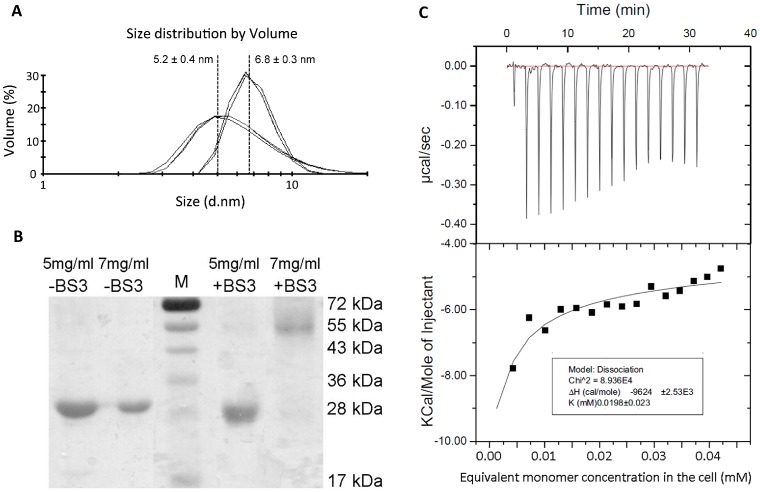
Analysis of the oligomeric state of FIV p24 as a function of the protein concentration. A) Size distribution profiles as a function of the volume of FIV p24-His at 5 mg/ml (left) and 7 mg/ml (right), by Dynamic Light Scattering. The graph shows the superposition of three successive measurements from 12 runs at each concentration, which are representative for at least three independent experiments. B) Chemical cross-linking of recombinant FIV capsid protein with protein at 5 mg/ml or 7 mg/ml. Lane MW, protein molecular mass marker. A negative control without BS^3^ has been realized on the same proteins (−BS^3^). C) Dissociation of FIV p24 dimer as followed by ITC. The thermogram (top panel) and the plotted titration curve (bottom panel) were obtained with a Microcal ITC200. The solid line (bottom panel) represents the fitting of the data by the built-in dimer dissociation model.

The FIV capsid protein was analysed by DLS (Figure 3A) to determine its oligomeric state in solution at two different protein concentrations (5 mg/ml and 7 mg/ml).

In each case, a single peak was observed, confirming that the protein solution was homogeneous. The average diameter of the p24-His protein at 5 mg/ml, which was obtained from three successive measurements, was 5.2 nm with a standard deviation of 0.4 nm (Figure 3A). This value agreed with the monomeric form of FIV p24 [35]. In contrast, the protein at 7 mg/ml had a mean diameter of 6.8 nm with a standard deviation of 0.3 nm (Figure 3A). This diameter indicated that the protein was mainly in dimeric form at 7 mg/ml.

In order to verify the oligomeric state of proteins in solution as a function of the concentration, we performed cross-linking experiments of p24 protein at 5 and 7 mg/ml using a BS^3^ cross-linker. The FIV p24 protein was mainly monomeric at 5 mg/ml, which contrasted with the mainly dimeric form of the protein at 7 mg/ml visible in the presence, and not in the absence, of BS^3^ (Figure 3B). No higher molecular weight species were observed at the two concentrations tested. Thus, the oligomeric state of the FIV p24 protein was concentration-dependent, shifting from monomeric at 5 mg/ml to dimeric at 7 mg/ml. To further investigate the nature of the dimeric form of p24, isothermal titration calorimetry (ITC) was performed using the protein at 7 mg/ml (Figure 3C). This experiment showed a single-step dissociation with an estimated dissociation constant Kd of around 20 µM.

### Monitoring of Capsid Assembly by Dynamic Light Scattering

For HIV, *in vitro* capsid assembly can be achieved by the modification of the ionic strength of the protein environment. The formation of particles during capsid assembly is followed by an increase in light scattering that can be easily detected [30]. DLS measurements of the FIV recombinant p24 protein were made in different conditions to monitor the *in vitro* capsid assembly. We tested the ability of the FIV p24 protein to assemble in various conditions of salt concentration, incubation temperature, and protein concentration.

**Figure 4:**
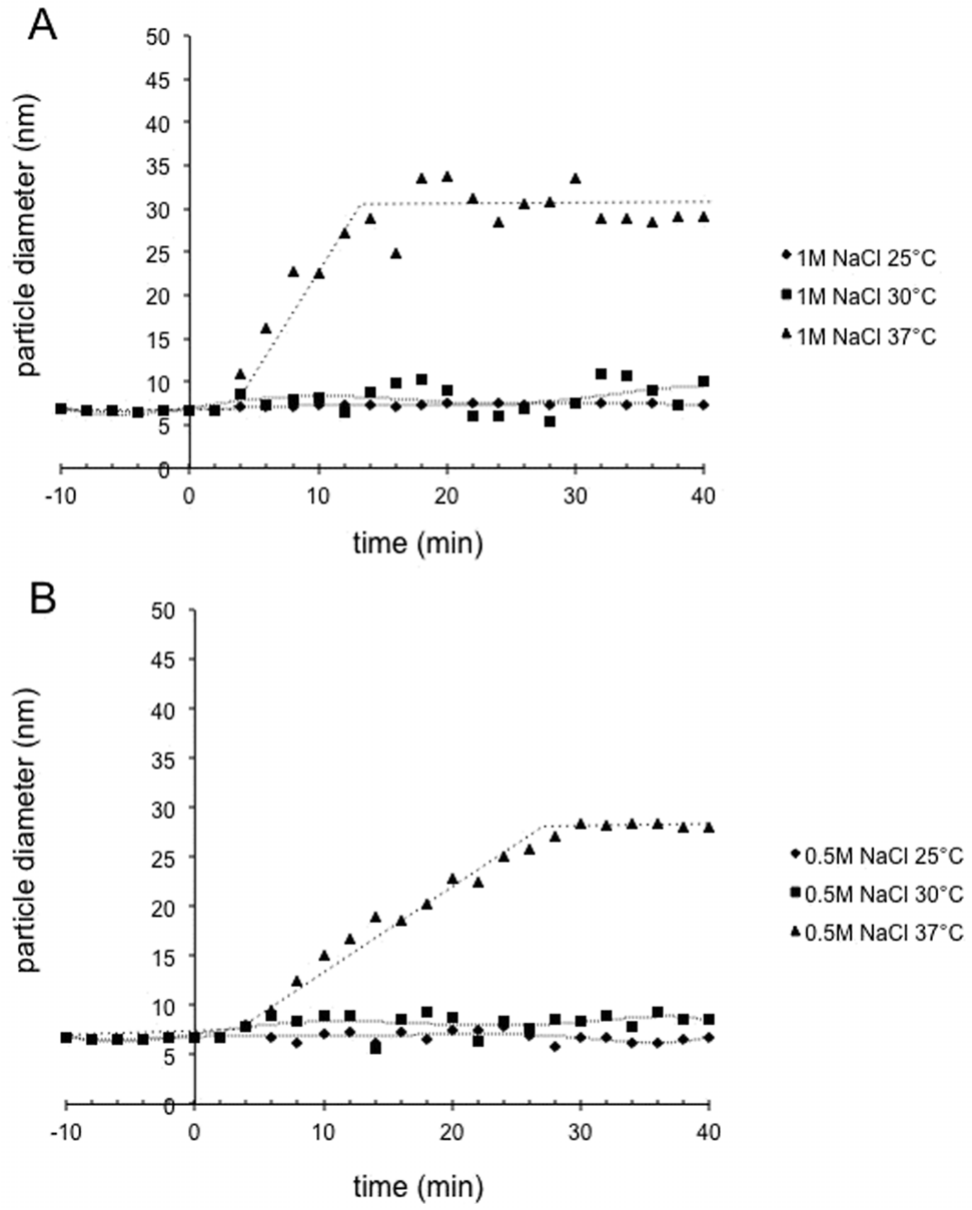
Monitoring of FIV capsid assembly by DLS with varying salt concentration and incubation temperature. T = 0 indicates the addition of NaCl to the protein solution at 7 mg/ml. Two NaCl induction conditions were tested: A) 1 M and B) 0.5 M NaCl (final concentration), with three different incubation temperatures: 37°C (triangles), 30°C (squares), and 25°C (diamonds).

The first assembly experiment was performed at 37°C using a protein sample at a concentration of 7 mg/ml in 1 M NaCl buffer (see method) (Figure 4A). We controlled the diameter before the addition of NaCl to the solution. The average diameter remained constant around 7 nm for 10 min at 37°C (Figure 4A). This matched the dimeric protein at 7 mg/ml (Figure 3). After the addition of 2 M NaCl buffer (1 M final NaCl concentration) at 37°C, the mean particle diameter increased steadily in solution. This probably reflected a change in the oligomeric state of the protein. During incubation, the protein moved to a higher form of oligomerization, reaching a final average diameter of 32 nm. A plateau was reached in less than 15 min, when the diameter appeared to stabilize. We then compared the assembly of the p24 at 7 mg/ml as a function of the salt concentration added (Figure 4B). With a lower final NaCl concentration (0.5 M) at 37°C, assembly took place with much slower kinetics than with 1 M salt concentration. The plateau was reached 30 min after increasing the final salt concentration to 0.5 M, whereas it was reached in less than 30 min with a final 1 M NaCl concentration (Figures 4A and 4B). Estimating the assembly rate from the slope of the linear part of each curve, the assembly in the presence of a final concentration of 0.5 M NaCl was ∼35% slower than with 1 M NaCl. The salt concentration had an effect on the rate of assembly, but at later time points (40 min) the final diameter of particles at the plateau was independent of the salt concentration. This suggests that an increased salt concentration increased the rate of assembly, rather than the amount of FIV p24 protein involved in the assembled objects.

We then tested the effect of temperature on p24 assembly. Assembly could only be detected at a physiological temperature of 37°C and no assembly was detectable at 25°C or 30°C, using either 1 M or 0.5 M NaCl (Figures 4A and 4B, respectively). Thus, temperature was a critical parameter in the experiment, in contrast to the *in vitro* assembly of HIV p24 proteins.

**Figure 5:**
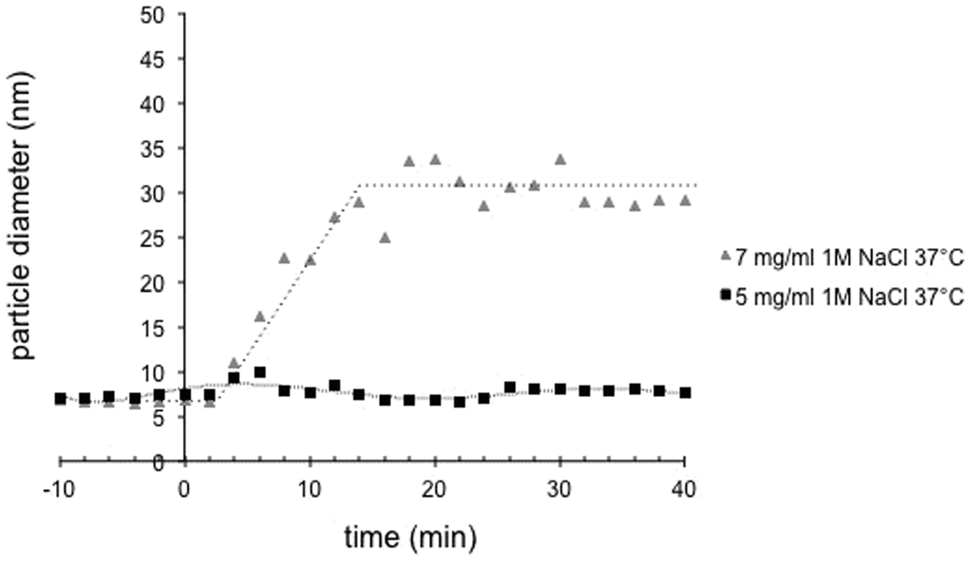
Monitoring of the FIV capsid assembly by DLS with varying protein concentration. T = 0 indicates the addition of 1 M NaCl to the protein solution at 7 mg/ml (gray triangles) or 5 mg/ml (black squares) at 37°C.

Finally, to evaluate the effect of protein concentration on the assembly kinetics, we tested two initial concentrations of p24 protein, i.e., 5 and 7 mg/ml. The assembly was performed at 37°C with the addition of 1 M final NaCl concentration. Assembly was not observed using the protein at 5 mg/ml (Figure 5) whereas assembly occurred at 7 mg/ml. Thus, the initial protein concentration also appeared to be a crucial parameter determining *in vitro* assembly.

In conclusion, the rate of FIV p24 assembly was dependent on the salt concentration, protein concentration, and incubation temperature. Based on these results, experiments were performed at 37°C with a final p24 protein concentration of 3.5 mg/ml in the remainder of the study.

### Morphology of Structures Formed by the Recombinant FIV Capsid Protein *in vitro*

To characterize capsid assembly, transmission electron microscopy (TEM) experiments were performed to visualize the assembled particles that were formed *in vitro*. As capsid assembly was rapidly induced at 37°C by the addition of 1 M final of NaCl as examplified by the diameter measured by DLS multiplied two-fold within 5 min (Figure 4A), we conducted a TEM study after mixing p24 proteins with 0.5 M final NaCl concentration at 37°C, in order to slow down the assembly kinetics and visualize any possible intermediate forms. To avoid missing assembly intermediates, samples were not centrifuged before staining.

**Figure 6:**
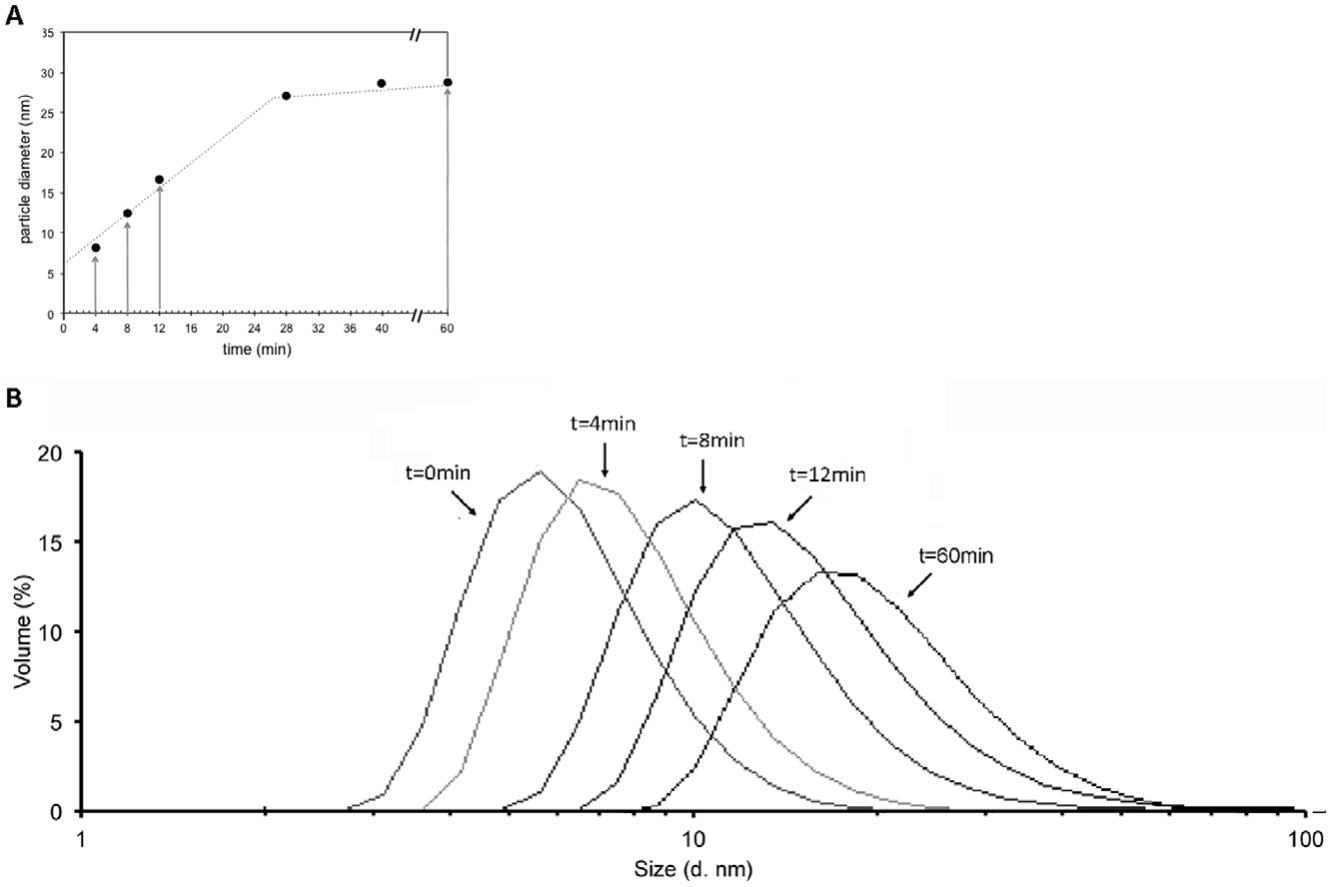
DLS experiments showing the kinetics of spherical assembly for p24 FIV. A) The assembly kinetics of the FIV p24 particles were monitored by dynamic light scattering with an initial protein concentration of 7 mg/ml and 1 M of NaCl at 37°C. Arrows indicate the time points when samples were taken for TEM analysis. B) Size distribution profiles as a function of the volume of FIV p24-His at different time points during assembly by DLS. Sampling times are indicated above their respective peak.

**Figure 7:**
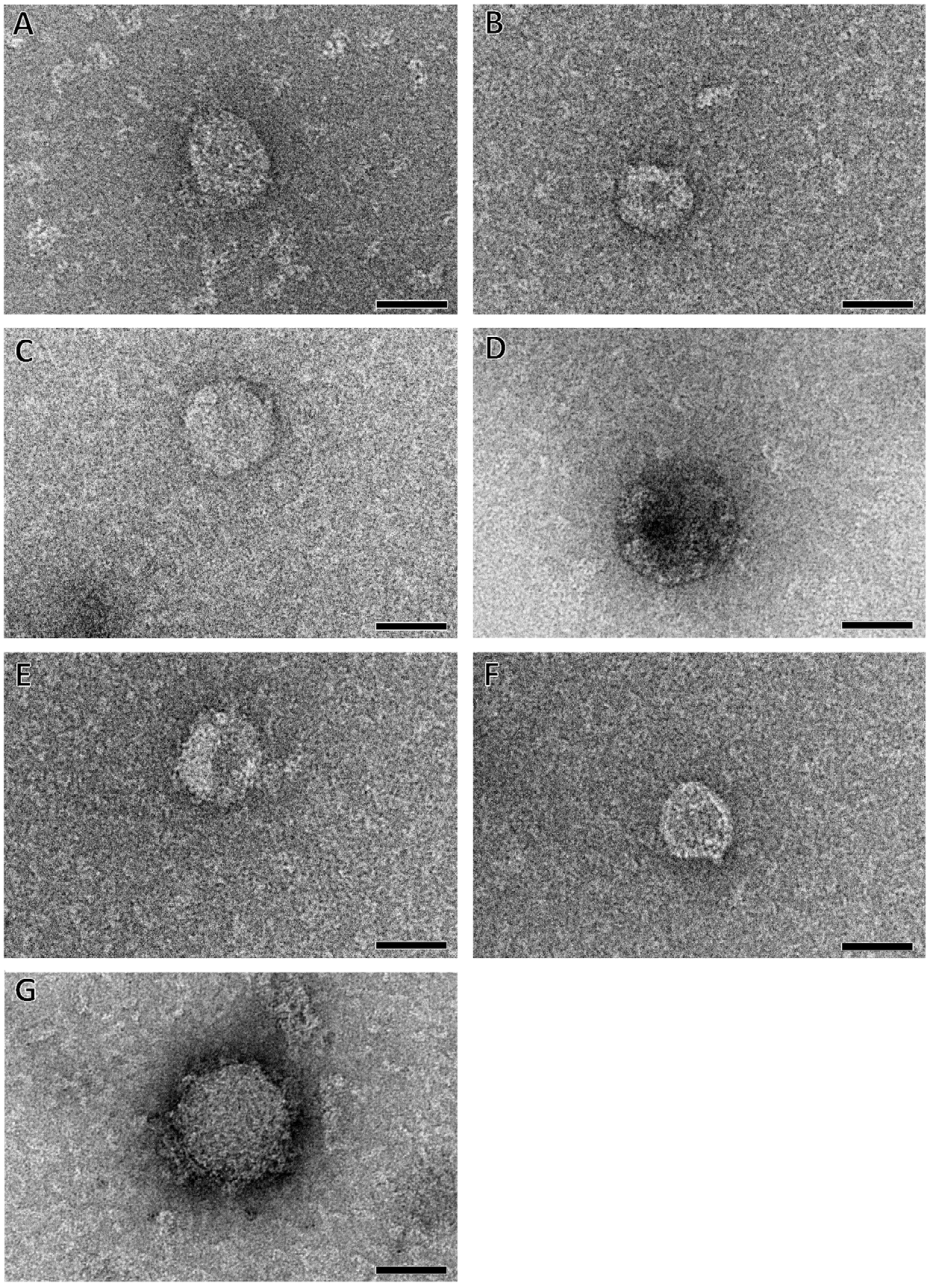
Kinetics of p24 assembly as shown by negatively stained TEM. Images taken after 4 min (A, B), 8 min (C, D), 12 min (E, F) and 60 min (G) of incubation, representative of 5 different assembly experiments. The samples were negatively stained with 2% ammonium molybdate. Magnification and bars, 45,000× and 50 nm.

Different assembly times in the exponential phase were sampled at 0 min, 4 min, 8 min, 16 min and 60 min (Figure 6A), with mean diameters measured by DLS of 6.6±0.2, 8.1±0.1, 12.2±0.3, 16.6±0.1, and 28.5±0.4 nm, respectively. DLS peaks were broad, reflecting the possible presence of objects of different sizes (Figure 6B). Therefore, TEM analysis of the assembled objects was performed (Figure 7). Before the addition of NaCl, only protein aggregates could be observed (data not shown). After only 4 min of assembly in 0.5 M final NaCl buffer, spherical particles appeared (Figures 7A & B). The overall shape of the visible particles and their size distribution, varying from 40 to 80 nm, was similar throughout the assembly period (Figures 7C to 7G). Thus, the overall increase of diameter observed by DLS resulted from an increase with time in the number of assembled objects.

## Discussion

After the optimization of FIV p24 overexpression and purification protocols, and the biophysical analysis of the purified protein by DLS and circular dichroism, we used DLS to monitor the *in vitro* assembly of the FIV capsid protein induced by the addition of salt. We found that the recombinant FIV p24 protein could form stable high-order oligomeric structures under specific *in vitro* conditions. This assembly appeared to be highly dependent on the protein concentration, salt concentration, and incubation temperature. The assembly of isolated FIV p24 protein was not due to the presence of nucleic acids (which were absent from our protein preparation) by opposition to FIV Gag polyprotein [36], probably because of the absence of the nucleocapsid region in our construct.

Electron microscopy is frequently employed for the accurate qualitative characterization of assembly [10], [11], [14], [15], [17], [26], [32], [33], [37]–[46], although the quantification of TEM results is problematic and relatively expensive. The efficiencies of capsid assembly reactions can also be monitored by turbidity assays [30], although this method does not distinguish assembled structures from non-specific aggregates. In this study, we developed a quantitative monitoring method for FIV capsid assembly based on DLS. We showed that the assembly rate is dependent on the protein and salt concentrations, and the incubation temperature. The rate of assembly was also highly dependent on the protein concentration, suggesting that the rate-limiting step in assembly is the self-association of several molecules, as previously shown for *in vitro* HIV capsid assembly [30]. Thus, a critical concentration of protein may be required for the assembly of FIV p24, in contrast to HIV capsid assembly where a two-fold change in protein concentration results in a 16-fold increase in the rate of assembly and where assembly can be observed at both high and low concentrations [30]. Noteworthy, FIV p24 is monomeric at 5 mg/ml and dimeric at 7 mg/ml. Because *in vitro* assembly occurs only at the latter concentration, our results suggest that only dimeric FIV p24 proteins can oligomerize into particles and that p24 dimers are the basic subunit of higher-order oligomers. Previous studies have also shown that dimerization of the HIV capsid protein is required for *in vitro* assembly [30]. Multimerization could have an important role in promoting the *in vitro* assembly of capsid proteins, because it occurs naturally before the maturation of the virus. In fact, biological multimerization of Gag polyproteins, and therefore capsid domains, is essential for the formation of a structural framework for the immature virus particle. HIV-1 p24 exists in a reversible monomer-dimer equilibrium at neutral pH, and the major determinants of Gag multimerization are located in the CTD of the capsid protein, particularly its capacity to form dimers [47]. In contrast, RSV p24 remains monomeric at neutral pH but dimerization as well as *in vitro* assembly are induced by mild acidification [48], [49] or by the addition of anions such as phosphate [50]–[52]. Thus, it seems that the dimerization of the capsid protein is a general prerequisite for *in vitro* retroviral capsid assembly.

Interestingly, the dissociation constant of the FIV p24 dimer is rather low (∼20 µM) in the same order of magnitude than HIV capsid protein dimer-to-monomer dissociation (between 13 and 40 µM) as measured using analytical ultracentrifugation [28], [29], [53]. It should also be noted that the dilution of the FIV p24 protein by the addition of salt during *in vitro* assembly does not seem to induce a shift from a dimeric to a monomeric form, as exemplified by the DLS measurements that did not show a decrease in the diameter immediately after addition of NaCl (Figures 4 and 5). The dimer, when diluted with high concentrations of NaCl, will be submitted to an equilibrium between a dimer-to-oligomer assembly and a dimer-to-monomer dissociation. Because the dissociation constant is low, this equilibrium would be in the favor of the dimer to oligomer assembly, resulting in an immediate and steady increase of particle size, and the plateau would then be reached when no dimer is left in the solution. The rate of *in vitro* FIV capsid assembly was also dependent on the salt concentration. There are two explanations for this type of salt dependence: i) the presence of more salt can increase the rate of assembly directly by limiting unfavourable protein interactions; or ii) the increase in the salt concentration induces conformational changes within the p24 protein subunit, resulting in a higher fraction of protein that is functional for assembly. In both cases, the sensitivity of these higher-order structures to such factors indicates that polar interactions regulate formation on the assembly surface. Interestingly, this sensitivity of capsid assembly to salt concentration has been observed for both FIV (this manuscript) and HIV [30] but not RSV [34]. The fact that both FIV and HIV but not RSV assembly rely on polar interactions is coherent with the fact that FIV and HIV p24 proteins contain the same proportion of polar residues (24.7% and 23.9% respectively) while RSV capsid contains only 17.7% of polar residues.

Finally, we showed that temperature is a critical factor for *in vitro* FIV capsid assembly. In fact, the assembly of FIV p24 is only possible at 37°C, which is close to the feline physiological temperature of 38.5°C. In contrast, HIV assembly can occur at 4 or 5°C [16], [17], as well as at 20°C [30], while the number of assembled objects observed during the assembly reaction at 4°C increased four-fold when HIV samples were pre-incubated for as little as 15 min at 37°C [46]. Therefore, this temperature dependency seems to be specific for FIV capsid assembly *in vitro* compared to other retroviral capsids.

Overall, the *in vitro* assembly of the FIV capsid appears to depend on more stringent experimental conditions than the HIV capsid. This may reflect a requirement for precise protein/protein interactions that are sensitive to the environment. The elucidation of FIV p24 structure would help answering this point. Interestingly, the *in vitro* assembly occurred at a physiological temperature, suggesting that the protein/protein interactions we observed may also occur *in vivo*.

Using electron microscopy, we showed that the *in vitro* assembly of FIV p24 protein led to the formation of spherical structures. These spherical forms had a diameter ranging from 40 to 80 nm. This kind of size variation could be expected in the absence of symmetry in spheroids: unlike icosahedral viruses, HIV capsids should be viewed as a continuum of related structures rather than as a single unique assembly [54]. Moreover, such a size variation has already been documented for mature capsids of naturally assembled HIV virions which show a variation of about 100% in their capsid sizes, from 95 to 160 nm in length and from 45 to 105 nm in width [10]. This variation in size during viral replication is in the same range and the same order of magnitude than the variation that we observe reproducibly *in vitro*. DLS data suggested that the diameter of assembled particles *in vitro* was smaller, about 32 nm. This could be explained by the fact that the diameter by DLS is the average diameter of unassembled monomeric proteins, aggregates, and spherical particles in solution, leading to an underestimation of the actual diameter of the assembled objects, while the size of the object is directly measured by TEM. This would be coherent with the broad shape of the peak in DLS (Figure 6B). Another explanation, not exclusive to the first, could be that the assembled particles in solution may have been flattened during sample preparation on the continuous carbon film used for TEM. However, the HIV capsid protein are known to form spherical shells between 30 and 50 nm in diameter [6] and HIV Gag protein lacking the p6 domain also assembles *in vitro* into small particles of about 25–30 nm [40]. The VLP particles obtained with FIV Gag polyprotein in mammalian cells have a diameter of 130–150 nm [55], while the FIV particles assembled *in vitro* with the Gag polyprotein are smaller (33 nm in diameter) [36]. Therefore, our results are within the range of sizes observed for lentiviral spherical capsid assembly *in vitro*. It has to be noted that FIV capsid assembly is very fast as demonstrated by DLS, and we cannot exclude that we have missed assembly intermediates by TEM due to the time needed to process each sample. It might be interesting to study the kinetics of oligomerization by real-time monitoring techniques such as SAXS experiments.

In contrast to our observations during FIV assembly, the structures formed during *in vitro* HIV capsid assembly are mainly tubular [11], [16], [17], [26], [30], [32], [33], [38], [41], [45], [56]. This tubular assembly is thought to be representative of the protein/protein interactions involved in the mature viral particle. Spherical/cone-shaped measuring ≈ 30 to 50 nm in diameter have also been observed [16], [17], [32] and are described as representative of the interactions taking place in the immature particle. N-terminal [26], [32] and C-terminal [33] extensions of the HIV capsid protein, naturally present in the immature Gag polyprotein, have been suggested to be involved in these differences in morphology. In particular, the presence of a free proline at the N terminus of the CA protein after the maturation of the Gag protein is thought to be important for mature-like assembly through the formation of a N-terminal β-hairpin (absent in the immature Gag) that will stabilize this N-terminal region [57]. However, tubular assembly can also be obtained with native HIV CA capsid without an intact N-terminal β-hairpin [16]. Thus, the presence of a N-terminal β-hairpin does not seem to be the main determinant for tubular assembly of HIV capsid *in vitro* [56]. It might however be interesting to evaluate the influence of N- or C-terminal extensions of the FIV CA protein on the morphology and the kinetics of its assembly *in vitro*. In that view, our circular dichroism experiments showed that the percentage of α-helix in the FIV capsid protein was about 41%. This value, as well as the overall spectrum shape and intensity, are very similar to previous data obtained for FIV capsid protein with a C-terminal histidine tag [58]. Thus, the presence of extra aminoacids at the N-terminus (our data) or at the C-terminus [59] has no significant incidence on the protein fold and preserves the protein ability to self-assemble. Indeed, we obtained similar data with our construct in the absence of the N-terminal extension (data not shown).

Experimental reconstruction of FIV spherical particles by cryoelectron microscopy (cryo-EM) would certainly allow a better comparison of FIV p24 capsid assembly *in vitro* with that of published retroviruses. Previous applications of cryo-EM techniques have facilitated the visualization of retroviral cores, i.e., HIV conical structures [17] and RSV polyhedral shapes [34]. However, FIV capsid particles obtained after *in vitro* assembly were not sufficiently homogeneous in size and the amount of assembled objects was too small to perform cryo-EM experiments.

HIV capsid assembly is believed to consist of a slow nucleation period followed by a rapid phase of tube growth [46]. Flexible fibers could be readily formed and dissociated by adjustments of the HIV capsid protein concentration, the pH, or the ionic strength of the solution [42], [46]. During RSV assembly, spherical and icosahedral structures are formed only at low pH [34] and they match the authentic viral core [48]. Thus, although many retroviral capsids have a similar capsid assembly process *in vivo* (spherical and conical in the immature and mature virus, respectively), capsid assemblies are different *in vitro*. Given the low sequence identity between capsid proteins, these differences in functional assembly *in vitro* probably reflect physico-chemical differences among these proteins. Given that the structure of the HIV capsid protein is known [56], [60], it would be interesting to solve the three-dimensional structure of the FIV p24 protein and to compare their dimerization interface, thereby identifying any determinants that might explain their different *in vitro* assembly.

Structural data would also allow mutagenesis experiments to decipher the specificity of the FIV p24 interactions during assembly and to confirm whether or not our *in vitro* assembly system is relevant for the *in vivo* situation. The obtention of structural data for FIV p24, or at least its CTD domain (which is the main determinant of p24 oligomerization for HIV) will probably be the next step towards the understanding of FIV capsid assembly. Moreover, getting structural informations on FIV p24 and its dimeric interface would allow the rational design of peptidic inhibitors of FIV capsid assembly, as it has been described for HIV[21]–[23], [59], [61]–[65]. Such compounds could be screened as a first approach in our DLS-based monitoring system of *in vitro* capsid assembly.
